# DFT (density functional theory) studies on cycloisomerization of 15–membered triazatriacetylenic macrocycle

**DOI:** 10.3906/kim-2012-15

**Published:** 2021-10-19

**Authors:** Mansooreh MOVAHEDI, Nader ZABARJAD SHIRAZ, Ali EZABADI, Marjaneh SAMADIZADEH, Mohamad Reza TALEI BAVIL OLYAI

**Affiliations:** 1 Department of Chemistry, Central Tehran Branch, Islamic Azad University, Tehran Iran; 2 Department of Chemistry, South Tehran Branch, Islamic Azad University, Tehran Iran

**Keywords:** DFT (density functional theory), triazatriacetyle, cycloisomerization, Ene–Reaction, Pd, [2+2+2] reaction

## Abstract

The mechanism as well the stereochemistry of cascade** **cycloisomerization of 15–membered triazatriacetylenic macrocycle was investigated theoretically by using M062X/6–31+G(d,p) and M062X/LANL2DZ calculations. The results showed that the mechanism and outcome of the reaction depended on the absence and presence of a transition metal catalyst. So that, in thermal-induced condition, the reaction had to experience several suprafacial concerted reactions including Ene-reaction (DG^#^=35.38 kcal/mol), Diels–Alder cycloaddition (DG^# ^= 17.16 kcal/mol), and sigmatropic H-shift rearrangement (DG^# ^= 56.21 kcal/mol) to produce diastereoselective fused cis–tetracyclic aromatic bearing a pyrrole moiety by following kinetic considerations. Also, the [2+2+2] cycloaddition mechanism was neglected in thermal–induced conditions because of high activation free Gibbs energy (DG^# ^= 63.90 kcal/mol). In the presence of palladium catalyst, Pd(0) formed an adduct by coordinating to C = C bonds and decreased the DG^#^ of the process to 29.58 kcal/mol, and consequently provided a facilitated media for the reaction to follow the [2+2+2] to produce more stable fused tetracyclic benzenoid aromatic by passing through the lower energy barrier.

## 1. Introduction

**Scheme 1:**
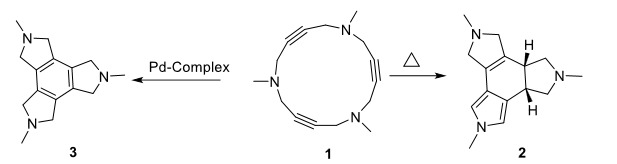


Cascade reactions are among reliable methods to create a fused ring system of various bioactive organic compounds [1–4]. Cycloisomerization of 15–membered triacetylenic azamacrocycle 1 is one of the interesting and powerful cascade reactions to synthesize tetracyclic fused rings in a one-pot reaction. The outstanding capability of this procedure is to provide four–membered fused rings with a desired stereochemistry (Scheme 1) [5]. To formulate these interesting structures, several attempts have been employed to follow the generality of the cycloisomerization of (**1**), including thermal-induced condition, the addition of auxiliary diene like cyclohexadiene, using azamacrocycle bearing phenyl substituent, utilizing transition metals like Pd, Co, Ru, and Cu complexes [6–17].

**Scheme 2:**
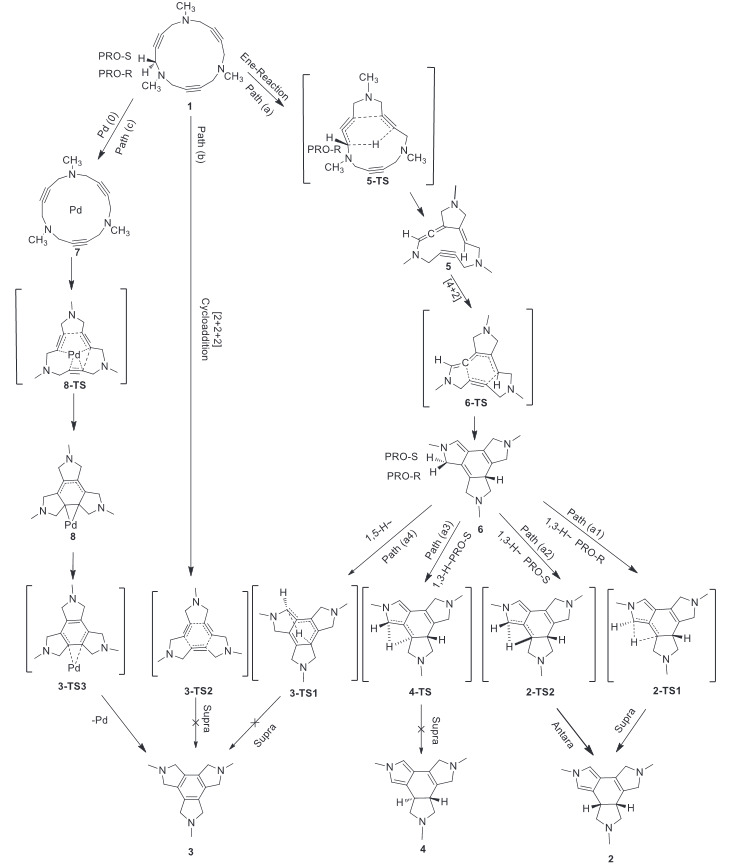


Based on published results, the thermal-induced reaction including 15-membered triacetylenic azamacrocycle (1) constructed tetracyclic fused ring 2 having outstanding diastereoselectivity (> 99:1 dr). There are some studies to explore the mechanism of cycloisomerization of macrocycle 1. In the most reliable representation for the thermal-induced process, a proposed mechanism included a cascade intramolecular Ene, then Diels–Alder reaction, and finally sigmatropic H-shift steps (Scheme 2) [5]. According to the published DFT calculations, a water molecule was needed to facilitate hydrogen migration [7]. However, experimental results confirmed that the reaction could happen in refluxing toluene as a nonaqueous solvent [5].

In the presence of Pd, compound 1 was converted to fused tetracyclic benzenoid aromatic 3 [11]. To expand the role of transition metals, several catalysts have been utilized to lead the outcome and stereochemistry of this reaction. Pd and Co complexes have been common catalysts for this purpose. In one successful challenge, 1 was treated with stoichiometric quantities of Palladium complex Pd(PPh_3_)_4_ in boiling toluene to produce triazaindane 3 [9,11]. Besides, the stable Pd(0) macrocycle complex was formed in THF at ambient temperature in which the three triple bonds of 1 have participated in complexation with Pd [18]. Although these metals have promoted and affected the outcome of this reaction, the literature showed that using Ru complexes improved the yield of the reaction significantly [19].

Recent increasing interest in cycloisomerization of 15-membered triacetylenic azamacrocycle, along with inadequate data about the explanation of the stereochemistry of this cycloisomerization, encouraged us to study the mechanism and diastereoselectivity of cascade cycloisomerization of 15-membered triacetylenic azamacrocycle reaction [5]. In addition to explain the diastereoselectivity of the cycloisomerization of azamacrocycle (1), we try to explain whether the reaction is controlled kinetically and/or thermodynamically in thermal-induced condition and also in utilizing of Pd(0) catalyst. 

## 2. Calculations

**Figure 1:**
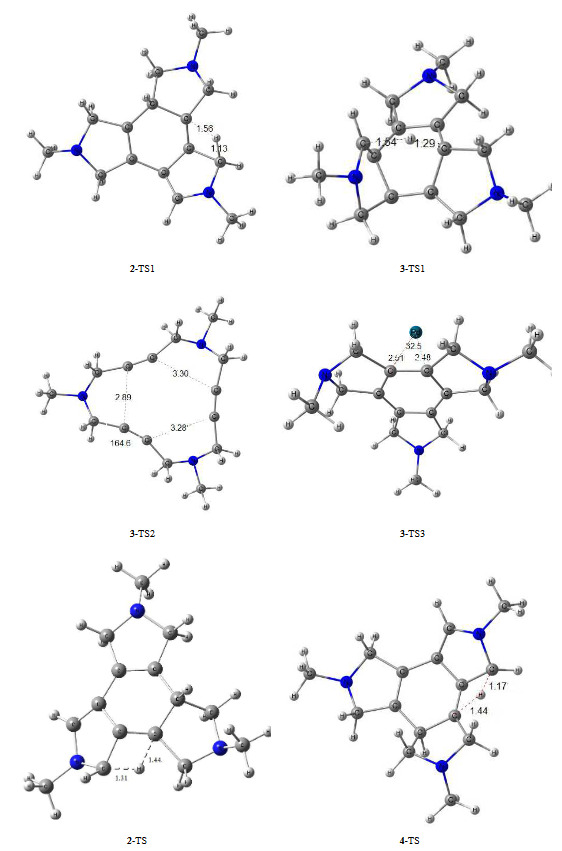

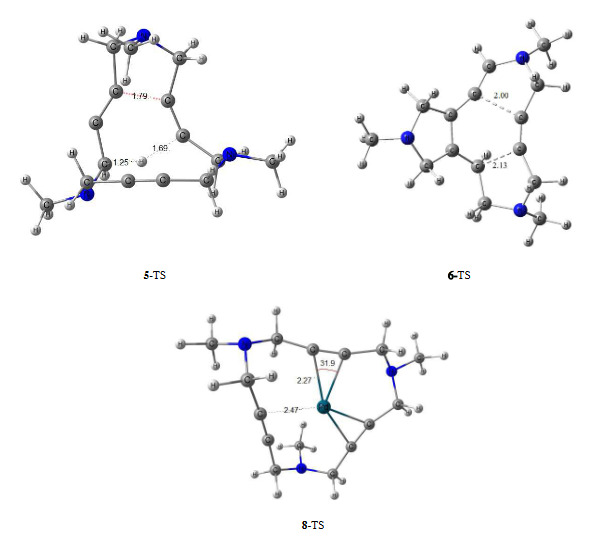
Optimized structures of transition states involved in mechanisms (Bond lengths in Å)

**Figure 2:**
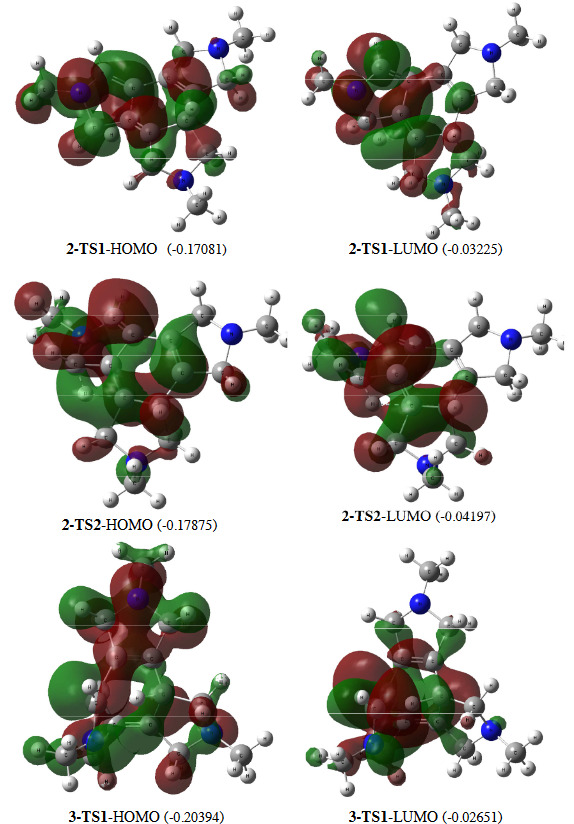

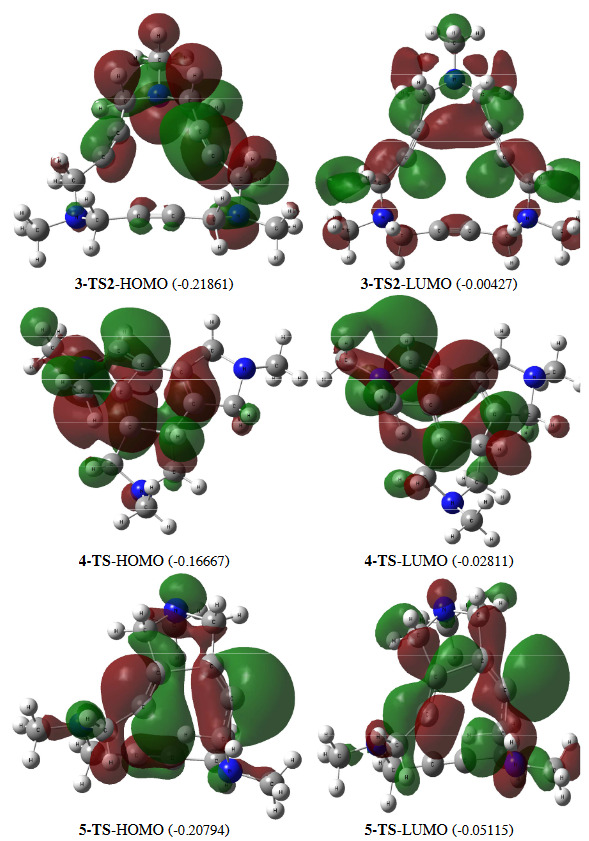

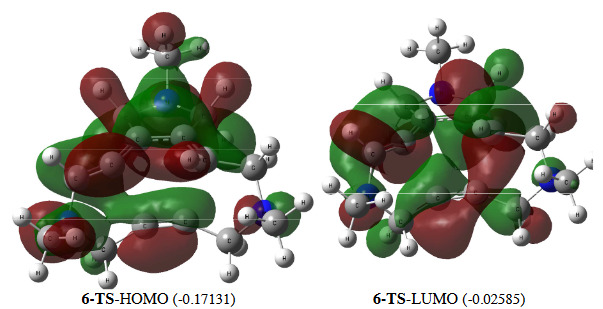
HOMO and LUMO illustration and energy (eV, in parenthesis) for transition state structures

The optimized structures were obtained without applying any constraints by using the LANL2DZ for Pd atom and 6-31+G(d,p) basis set to describe the electrons of C, H, N atoms using density functional theory method (M062X) provided in Gamess software [20]. Frequency analysis were used to confirm if the optimized structure was a global ground state or a transition state structure. So that, reactants, intermediates, and products possessed zero imaginary frequency while transition states had only one imaginary frequency. In addition, frequency calculation provided zero-point energies (ZPE) as well other thermodynamic data including Gibbs free energy [21]. To verify the structures of transition states, IRC calculations were utilized to relocate starting material and products from the optimized transition states. To provide energy profile of the reaction, the Gibbs free energy (in a.u. or Hartree) of starting material 1 was considered as zero, and the relative Gibbs free energies (in kcal/mol) of the other components including products (2-4), intermediates (5 and 8) and transition states (5-TS, 8-TS) were compared to the energy of compound 1. The results of calculated electronic energy, zero point energy, Gibbs free energies (Table) are presented in the supporting file along with the Cartesian coordinates of optimized geometry of each structure (Figure 1). The NBO analysis was performed for transition sate structures at the same level of theory to calculate energies and obtain shapes of the HOMO and LUMO orbitals. The energy (ev) and the shape of frontier orbitals (Figure 2) and structures (Figure 1) were represented in supporting file.

**Table:** Electronic energy (EE), zero point energy (ZPE), Gibbs free energy (G), and relative stabilities (DG, compared to 1).

Comp.	EE (a.u.)	ZPE (a.u.)	G (a.u.)	DG (kcal/mol)
1	–747.965482	0.337400	–747.671933	0.00
5-TS	–747.905201	0.333848	–747.615545	35.38
5	–748.008715	0.338610	–747.714063	–26.44
6-TS	–747.979690	0.335177	–747.686714	–9.28
6	–748.131753	0.342567	–747.833443	–101.35
3-TS1	–748.003912	0.338293	–747.723712	–32.49
3	–748.175562	0.341655	–747.875639	–127.83
2-TS1	–748.009503	0.340906	–747.743871	–45.14
2	–748.165432	0.343031	–747.864641	–120.93
2-TS2	–747.9940841	0.3379833	–747.691537	–12.30
4-TS	–747.986090	0.339576	–747.704899	–20.69
4	–748.158857	0.343741	–747.858498	–117.07
3-TS2	–747.864843	0.337301	–747.570095	63.90
7	–874.613883	0.338154	–874.324775	–10.23
8-TS	–874.5540239	0.338165	–874.26164	19.35
8	–874.711530	0.344535	–874.412431	–75.27
3-TS3	–874.684006	0.342284	–874.389114	–60.64

## 3. Results and discussion

Experimental research showed that the cascade cycloisomerization of 15-membered triacetylenic azamacrocycle (1) resulted in diastereoselective fashion (de>99%) (Scheme 1) [5]. To explain the observed diastereoselectivity, theoretical studies performed on starting materials, transition states, intermediates, and products to provide energy profile of the reaction. 

A number of opportunities were available for 15-membered triacetylenic azamacrocycle (1) to perform cycloisomerization in the mentioned reaction. Among several potential mechanisms, experimental studies rejected radical involvement in the mechanism [19]. Based on published documents, three distinct proposal mechanisms (path (a), path (b), and path (c)) were represented in Scheme 2 [7, 18]. In the current project, each pathway was designed individually and all structures involved in the mechanism were optimized at M062X/6–31+G(d,p) and M062X/LANL2DZ levels of theory. The relevant energies of optimized components were summarized in Table imbedded in supporting file. 

*Path (a)*: This path represented a domino process including three concerted phases. The reaction was started by an Ene-reaction, followed by a [4+2] cycloaddition, and finalized by a sigmatropic H-shift which led to the formation of cyclohexadiene tetra-fused ring 2, diastereoselectively (Scheme 2). The first step afforded an allene group in intermediate 5, which was ready to perform a pericyclic [4+2] cycloaddition with C≡C bond to give a 1,4-cyclohexadiene (6). The last compound (6) went through a hydrogen shift to yield products 2-4. The major diastereoselective product (2) contained a pyrrole moiety attached to a cis–cyclohexene center and a saturated azacyclopentyl ring fused to cyclohexene ring (Scheme 2) [5]. The mentioned sigmatropic H-–shift step was responsible to induce and control the stereochemistry of product 2. 

Optimized structures showed that compound 1 was not flat and had a quasi–boat shape structure. The two triple bonds were almost planar, while the next triple bond made 117˚ angle with mentioned imaginary plane. In the optimized 5-TS structure, the migrating hydrogen was located at 1.255 Å from the starting carbon atom and 1.691 Å from the target carbon atom, and the new forming C-C length was 1.798 Å. This optimized structure was almost the same structure which has been reported in the presence of a water molecule [7]. In the optimized allene structure (5), the C=C bond length was 1.313 Å and the two C=C bonds were perpendicular to each other. In 6-TS, which was a transition state for the Diels–Alder reaction, distance between interacting carbon atoms were 2.007 and 2.139 Å. In 3-TS1 the migrating hydrogen atom was located at 1.542 and 1.292 Å far from the departing and destination carbon atoms. In 2-TS1, the migrating Pro-R hydrogen was closer to the primary carbon (1.136 Å) rather than the target carbon (1.566 Å). In the optimized structure of 4-TS, the Pro–S hydrogen was at 1.441 and 1.176 Å far from the corresponding carbon atoms, respectively (Figure 1).

The overall reaction was exothermic (DG = –120.93 kcal/mol). The mechanism was initiated by interaction of acetylenic carbon–carbon bonds at 5-TS (Figure 1). The process from 1 passed through the 5-TS (DG^# ^= 35.38 kcal/mol) to give the vinylallene 5, which was a critical material to perform the next concerted cycloaddition step. This DG^# ^was slightly greater than the previous reported one (DG^# ^= 30.1 kcal/mol, B3LYP/cc-pVDZ ) [7]. Based on HOMO orbital of 5-TS, the transferring hydrogen and the target carbon of alkene moiety were in the same phase having an effective overlap. On the other hand, the orbital located on three carbon atoms of the allenic system were in the same phase and displayed sufficient overlap (Figure 2). So, a suprafacial orbital interaction in the 6-electron pericyclic reaction provided a constructive sigma shift to produce allene 5. 

The 6-TS structure was considered as a transition state for Deals-Alder reaction which led to the formation of 6 (Scheme 2). The [4+2] cycloaddition step overcame the barrier of 6-TS (DG^# ^= 17.16 kcal/mol) and formed compound 6, which was 101.35 kcal/mol more stable than compound 1. Thus, under thermal condition, this step of the reaction could be considered irreversible. 

As the HOMO orbital displayed, the asymmetrical same phase orbital has been located on 1 and 4 carbon atoms of diene. Also, this orbital was in the same phase with the orbital positioned on two carbon atoms of dienophile moiety. Therefore, the shape of HOMO orbital has provided a proper supra-–supra facial overlap of diene and dienophile, consequently has projected an asymmetric polar Deals–Alder reaction. So that, this step started with approaching the allene carbon to dienophile, and followed by attaching another carbon of diene to the next carbon of dienophile. In addition, the LUMO orbital, showed a constructive overlap between one carbon of diene and a carbon atom on dienophile part.

To continue the reaction, compound 6 performed a sigmatropic H–shift to produce more stable compound 2. There were two hydrogen atoms (Pro-R and Pro-S) available to perform suprafacial or antarafacial H-shift to produce 2-4, hypothetically. Suprafacial Pro-R hydrogen migration would produce 2 (Path a1), while the suprafacial Pro-S migration led to the 4 (Path a3). Also, Path (a2) was an antarafacial option for Pro-S migration to produce diastereomer 2. In addition, compound 3 could be formed via a suprafacial 1,5-H migration (Path a4) (Scheme 2).

Path (a1): The suprafacial 1,3-sigmatropic reaction of Pro-R hydrogen atom via 2-TS1 as a transition state resulted in the formation of diastereomer 2. The [1,3]-H sigmatropic step had to tolerate a relatively high barrier (2-TS1, DG^#^=56.21 kcal/mol). The instability resulted from internal strain of 4-membered ring in 2-TS1 was the most difficult challenge for the reaction to complete the processes. Consequently, this step has been considered as the rate-determining step of the reaction. 

In the HOMO orbital, the migrating hydrogen was in the same phase with the departing carbon but in the opposite phase to the destination carbon (Figure 2). Therefore, according to frontier orbital theory, in thermal conditions hydrogen atom preferred an antarafacial transfer to the other side of the molecule and produce structure 4. But geometrically, it was difficult to hydrogen to transfer from one side to the opposite side of a rigid molecule. Considering the LUMO orbital, the hydrogen and the target carbon were in the same phase providing constructive suprafacial overlap leading to formation of product 2. Although it is not a general idea, in this case LUMO orbital could explain the mechanism of conversion of 6 to final product 2.

Path (a2): Product 2 could be produced by antarafacial sigmatropic shift of Pro-S hydrogen atom via 2-TS2 transition state.

*Path (a3)*: Theoretically, there was another possibility for Pro-S hydrogen atom to produce trans-fused ring diastereoisomer (4) via suprafacial 1,3-sigmatropic reaction of 4-TS. However, this product has not been obtained experimentally. 

The shape and discussion for frontier orbitals of 2-TS2 and 4-TS was mostly similar to 2-TS1 (Figure 2). So that, based on frontier orbital theory, 2-TS2 preferred antarafacial migration to produce cis-isomer 2. As mentioned earlier, in terms of geometrical facility, migration to opposite side (antarafacial mode) of rigid molecule (6) has been a struggling process. 

HOMO and LUMO orbitals of 2-TS1, 2-TS2 and 4-TS showed that forecasting of diastereoselectivity based on the frontier orbitals theory was in contrast with kinetic control predictions and experimental findings. Kinetically, the DG^#^ energy of the 2–TS1 path to the formation of cis-isomer 2 was less than that of 4-TS to formation of trans-isomer 4. Experimentally, the reaction produced diastereomer 2 from the kinetic control pathway with a lower 2-TS1 activation energy. In other words, the molecular orbital method was not as straight forward as supposed to justify the diastereoselectivity (formation 2 instead of 4) of this reaction. In addition, constructive suprafacial overlap has been provided in LUMO orbital of 2-TS1, which meant photochemical condition would facilitate diastereoselectivity of the reaction to produce 2 as a final product.

**Figure 3:**
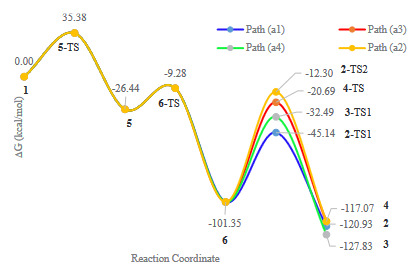
Relative stabilities (DG, in kcal/mol) of the intermediates and relevant transition states involved in path (a) mechanism compared to Gibbs free energy of 1.

The comparison of the energy profile of the formation of 2 with 4 showed that in this case, the energy barriers of 2-TS1, 2-TS2 and 4-TS were 56.21, 89.05, and 80.66 kcal/mol, respectively. This result explained the reason for the formation of predominant product 2, as a kinetically favored product (Figure 3) [5]. 

Path (a4): 3-TS1 represented an imaginary transition state structure to study the possibility of a 1,5-hydrogen sigmatropic reaction, which lead to the production of isomer 3. Based on the molecular orbital symmetry conservation rules, 1,5-sigmatropic H-shift is more favorable than a 1,3-sigmatropic shift from the molecular orbital point of view. But in the current circumstance, 1,5-sigmatropic H-shift was more difficult geometrically. The structure of compound 6 could explain the reason for this surprising observation. The planar conjugate part (sp^2^ carbons) of the molecule (6) must be folded in a transition state 3-TS1 to complete a concerted suprafacial 1,5-sigmatropic shift. Molecule 6 was rigid and it had not such a capability to bend the bonds properly to provide appropriate orientation to perform a 1,5-sigmatropic H-shift. As the structure of transition state 3-–TS1 indicated, the structure suffered from internal strain or angle strain. So that, the exocyclic double bond attached to the central ring was out of plane (33.2 ˚) and made this reaction challenging or impossible to happen (Figure 1). 

The HOMO orbital of 3-TS1 showed that it has not been located significantly on the migrating hydrogen and has a little overlap with the destination carbon. The LUMO orbital has located on the migrating hydrogen, and the destination carbon with the same phase showing a complete overlapped. So, low density of HOMO orbital on migrating hydrogen and difficulty of bending of planar structure could explain the lack of product 3 as the thermal-induced product of the reaction. 

A comparison of the relative stabilities of 2-4 showed that compound 3 having a benzenoid aromatic structure was the most stable isomer. But in practice, under thermal–induced conditions, this product had not been identified as a major product. In addition, 4 was more stable (2.65 kcal/mol) than the dominant product 2. In other words, the reaction did not follow the thermodynamical control while it has been under kinetic control.

The published theoretical studies emphasized the role of a water molecule, which increased the rate of reaction [7]. But, usually, there is not a water molecule involved in the mechanism, and the reaction was conducted in refluxing toluene; we tried to study the mechanism of the reaction in gas phase.

Path (b): In the concerted [2+2+2] pathway, the reaction took place by passing through 3-TS2 as a transitional state in which the forming carbon-carbon bonds had distance of 2.896, 3.288, and 3.304 Å from each other. In this case, the acetylene groups deviated from the linear C-C≡C model by the angle of 164.6°.

**Figure 4:**
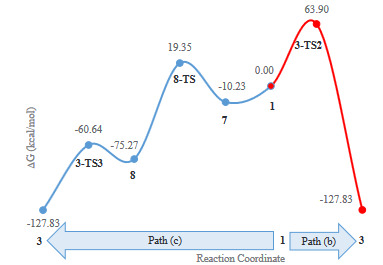
Relative stabilities (DG, in kcal/mol) of the intermediates and relevant transition states involved in path (b) and path (c) mechanisms compared to Gibbs free energy of 1.

In this mechanism, the reaction had to overcome the barrier of 63.90 kcal/mol to produce 3 (Figure 4). This barrier was higher than the activation free energies of other proposed pathways, and experimental studies have not verified the formation of isomer 3 in a thermal-induced [2+2+2] reaction or isomerization of 2 to 3.

HOMO orbital of 3-TS2 showed that this orbital has not been distributed symmetrically over the π system of transition state. In addition, this orbital was not in suitable orientation to perform head-to-head constructive overlap and consequently, did not deliver a C-C sigma bond formation. Therefore, from the frontier molecular orbital point of view, there was no appropriate overlap to construct a sigma bond and perform the [2+2+2] reaction to produce isomer 3. In addition, there was a (63.90 kcal/mol) to let the reaction to proceed further. This obstacle and relatively high activation Gibbs free energy of this step accumulated to prevent [2+2+2] cycloaddition reaction to happen successfully.

Path (c): Triaza macrocyclic scaffold of 1 has been converted into tetracyclic structure 3 performing a [2+2+2] cycloaddition reaction in the presence a Pd–catalyst (Scheme 2) [5]. Calculations showed that, according to the proposed mechanism, an adduct (7) was formed by the addition of Pd to compound 1, which was 10.23 kcal/mol more stable than starting material 1. This adduct (7) converted to intermediate 8 by passing through 29.58 kcal/mol energy barrier of 8-TS. Compound 8 was 75.27 kcal/mol more stable than 1 having more sigma bonds compared with 1. In 8, Pd was coordinated to the 6-membered central ring (Figure 1). In the next step, it crossed over the energy barrier of 14.63 kcal /mol, released Pd, and converted to 3 (Figure 4).

The optimized structure of adduct 7 was almost flat, which was in agreement with the experimental structure obtained from the reported X-Ray studies [18, 22, 23]. In this structure, the palladium was located at an approximate distance of 2.2 and 2.5 Å from the carbons of the triple bonds, and the triple bonds were slightly bent towards the palladium so that the C-C≡C angle was 165.0 °. In the 8-TS structure, the palladium was coordinated to three triple bonds. In this structure, the two triple bonds were closer to palladium (2.271 Å and C-Pd-C angle 31.90 °) and Pd was 2,476 Å away from the other triple carbon–carbon bond). In 3-TS3, where the central 6-membered ring was formed, the palladium was located on one of the C-C bonds of the 6-membered ring with a bond length of 2.510 and 2.481 Å and the C-Pd-C bond angle of 32.5 °.

## 4. Conclusion

Density functional theory calculations delivered a geometry pattern of materials participated in describing stereochemistry of cascade multicomponent reaction leading to stereoselective cycloisomerization of 15-membered triacetylenic hetero macrocycle. Calculated results along with experimental findings verified that the mechanism and outcome of the reaction depended on the absence and/or the presence of transition metal catalyst. So that, the catalyst formed an adduct by coordinating to carbon-carbon triple bonds and consequently provided a facilitated media for the reaction to follow the [2+2+2] cycloaddition to produce more stable fused tetracyclic benzenoid aromatic 3. In the absence of catalyst, the reaction had to experience several concerted reactions, including suprafacial Ene-reaction, supra-supra facial Diels–Alder cycloaddition, and constructive suprafacial sigmatropic Pro-R H-shift to produce diastereoselective fused tetracyclic aromatic 2 bearing pyrrole moiety, kinetically. Also, calculations showed that the [2+2+2] cycloaddition mechanism was neglected in thermal-induced conditions. It is worth mentioning that efforts to trap or separation of intermediates involved in the proposed mechanism and conducting the reaction in photochemical condition would be an opportunity for coming researches. This theoretical forecast may offer perceptions for researchers to provide innovative products for cascade cycloisomerization of 15-membered macrocycles and its derivatives especially designing anticipated stereochemistry.
